# Pharmacokinetic and Biomarker Quantification Studies on Vancomycin-Loaded PEGylated Liposomes and Its Potential to Reduce Vancomycin-Induced Kidney Injury: A Rat Study

**DOI:** 10.3390/pharmaceutics15061582

**Published:** 2023-05-24

**Authors:** Medha D. Joshi, Paulina Iacoban, Marc H. Scheetz

**Affiliations:** 1 College of Pharmacy, Midwestern University, Glendale Campus, 19555 N. 59th Avenue, Glendale, AZ 85308, USA; 2 College of Pharmacy, Midwestern University, Downers Grove Campus, 555 31st Street, Downers Grove, IL 60515, USA

**Keywords:** vancomycin, liposomes, nephrotoxicity, kidney, KIM-1, pharmacokinetics

## Abstract

Vancomycin is a commonly used antibiotic in hospital settings, especially against Methicillin-resistant staphylococcus aureus (MRSA). One of the major adverse events of vancomycin use in adults is kidney injury. The drug concentration, specifically the area under the concentration curve, predicts kidney injury in adults receiving vancomycin. To attempt to reduce vancomycin-induced nephrotoxicity, we have successfully encapsulated vancomycin in polyethylene glycol-coated liposomes (PEG-VANCO-lipo). We have previously carried out in vitro cytotoxicity studies on kidney cells using PEG-VANCO-lipo and found it to be minimally toxic compared to the standard vancomycin. In this study, we have dosed male adult rats with PEG-VANCO-lipo or vancomycin HCl and compared plasma vancomycin concentrations and KIM-1 as an injury biomarker in rat urine. Male Sprague Dawley rats (350 ± 10 g) were administered vancomycin (*n* = 6) or PEG-VANCO-lipo (*n* = 6) 150 mg/kg/day for three days using an IV infusion in the left jugular vein catheter. Blood was collected for plasma at 15, 30, 60, 120, 240, and 1440 min after the first and the last IV dose. Urine was collected 0–2, 2–4, 4–8, and 8–24 h after the first and the last IV infusions using metabolic cages. The animals were observed for three days after the last compound administration. Vancomycin was quantified in plasma by LC-MS/MS. Urinary KIM-1 analysis was done by using an ELISA kit. Three days after the last dose, under terminal anesthesia with IP ketamine (65–100 mg/kg) and xylazine (7–10 mg/kg), rats were euthanized. Vancomycin urine and kidney concentrations and KIM-1 were lower on day three in the PEG-Vanco-lipo group compared to the vancomycin group (*p* < 0.05, ANOVA and/or *t*-test). There was a significant reduction in plasma vancomycin concentration on day one and day three (*p* < 0.05, *t*-test) in the vancomycin group compared to the PEG-VANCO-lipo group. Vancomycin-loaded PEGylated liposomes resulted in lower levels of kidney injury, as noted by a decrease in KIM-1 values. Moreover, longer circulation in plasma with increased concentration in plasma as opposed to the kidney was observed with the PEG-VANCO-lipo group. The results indicate the high potential of PEG-VANCO-lipo in decreasing the nephrotoxicity of vancomycin clinically.

## 1. Introduction

Vancomycin is a commonly used antibiotic in hospital settings, especially against Methicillin-resistant staphylococcus aureus (MRSA) [1]. Patients infected with MRSA in critical care settings are likely to have co-morbidities such as chronic kidney disease [2]. Kidney injury is also a major adverse event of vancomycin in adults. Parameters such as the total vancomycin daily dose, duration of therapy, method of administration, trough level, and area under the concentration vs. time (AUC) curve are known to be potential risk factors for vancomycin-induced acute kidney injury [3]. Based on these factors, various approaches have been suggested to lower kidney injury. Lowering the AUC has been shown to decrease kidney injury [4,5]. 

Various biomarkers have been identified in the literature that can be applied to assess to quality and quantity of nephrotoxicity. We have previously identified the Kidney Injury Molecule-1 (KIM-1), whose expression is significantly up-regulated in the proximal tubule in the post-ischemic rat kidney, to be a highly sensitive and a very early marker, defining the onset of the kidney injury [6]. Moreover, we have developed a predictive model correlating urinary KIM-1 with the area under the curve (AUC) for predicting even a minimal level of histopathologic damage at 24 h of vancomycin dosing in a rat model [4,7,8,9]. KIM-1 is a type-1 transmembrane protein that is not normally present but is expressed on the proximal tubule apical membrane with injury [6]. Many studies have confirmed KIM-1 to be an extraordinary indicator of kidney injury in rats compared to the traditional blood urea nitrogen and serum creatine biomarkers as predictors of histopathological changes in the proximal tubule in response to kidney injury [10,11,12,13]. Additionally, the relationship between vancomycin AUC and KIM-1 is well established, with increasing AUC causing increased KIM-1 in the urine [7,8]. 

In an effort to reduce vancomycin-induced nephrotoxicity, we have successfully encapsulated vancomycin in polyethylene glycol-coated liposomes (PEG-VANCO-lipo) [14]. The particle size of PEG-VANCO-lipo was found to be less than 200 nm, and they were characterized for stability, zeta-potential change, and in vitro release kinetics, as reported in our earlier publication [14]. We have previously carried out in vitro cytotoxicity studies on kidney cells (rat kidney epithelial cells, NRK-52E) using PEG-VANCO-lipo and found it to be minimally toxic compared to vancomycin alone [14]. Liposomes are phospholipid bilayer structures. They are spontaneously formed with a resultant mean diameter of a few nanometers. The phospholipid bilayer structure can encapsulate water-soluble drugs, e.g., vancomycin, in its aqueous core. Encapsulation of drugs in liposomes has resulted in the reduction of nephrotoxicity for amphotericin B (AmBisome), which is the oldest and most commercial example of the use of liposome technology [15]. Moreover, encapsulation of antimicrobials in liposomes could offer enhanced pharmacokinetics (e.g., increased AUC) and pharmacodynamics and hence decreased toxicity [16]. 

Vancomycin has been encapsulated in various types of liposomes [17,18] using various methods of encapsulation [18] for the purpose of meeting various objectives [19,20,21,22]. These objectives include increasing its antimicrobial efficacy using fusogenic liposomes [23], targeting and enhancing the efficacy of its topical use [24], and use in pneumonia by increased deposition in lungs [25,26], etc. A coating of polyethylene glycol (PEG) provides the liposome particle a corona by which it can go undetected by the body’s defense mechanism via the reticuloendothelial system (RES), allowing circulation in the body for a greater amount of time compared to conventional liposomes that lack PEGylation. Hence, we hypothesized that a PEGylated liposome loaded with vancomycin (PEG-VANCO-lipo) would be able to circulate for a longer time in the body and avoid traditional glomerular filtration and associated kidney toxicity compared to vancomycin by itself. To the best of our knowledge, our method of preparation, thin-film hydration followed by the freeze–thaw method of PEG-VANCO-lipo with the objective of reducing nephrotoxicity and with a higher percentage of vancomycin encapsulated [14], is different from previously reported vancomycin-loaded PEGylated liposomes [25,26,27,28] and we are the first ones to report it. To test our hypothesis, we dosed male adult rats with PEG-VANCO-lipo or vancomycin HCl and compared their pharmacokinetic (PK) parameters and a highly sensitive kidney injury biomarker: KIM-1.

## 2. Materials and Methods

- Animals

Male Sprague Dawley (SD) rats weighing 270 ± 10 g were provided by BioLasco Taiwan (under Charles River Laboratories License). Space allocation for each individual animal was 47 × 25 × 21 cm^3^. All animals were maintained in a controlled temperature (20–24 °C) and humidity (30–70%) environment with 12 h light/dark cycles in Pharmacology Discovery Services Taiwan, Ltd. Laboratory (Taipei, Taiwan). Free access to a standard lab diet (Oriental Yeast Co., Ltd., Tokyo, Japan) and autoclaved tap water was granted. All aspects of this work, including housing, experimentation, and animal disposal, were performed in general accordance with the “Guide for the Care and Use of Laboratory Animals: Eighth Edition” (National Academies Press, Washington, DC, USA, 2011) in their AAALAC-accredited laboratory animal facility. In addition, the animal care and use protocol was reviewed and approved by the IACUC (number PK001–08242021) at Services Pharmacology Discovery Taiwan, Ltd. (PDST), (Taipei, Taiwan).

- Chemicals

The 0.9% NaCl (Sing-Tong, Taiwan), Acetonitrile (CAN; Fisher Scientific U.K. Ltd., Loughborough, England), Bupivacaine (Marcaine^®^; AstraZeneca, Cambridge, UK), formic acid (FA; Merck, Germany), DMSO (Merck, Darmstadt, Germany), Ketalar injection (50 mg/mL; Pfizer, New York, NY, USA), methanol (MeOH; AENCORE, Australia), PBS (Sigma, USA), pentobarbital (Health-Tech Pharmaceutical Co., Ltd., Taiwan), Rompun injection (2%; Bayer, Leverkusen, Germany), and sodium heparin bodene (Pty) (Limited Trading as Intramed, Maharashtra, India). Acetonitrile, chloroform, and methanol VWR International (Radnor, PA, USA). Formic acid and PBS pH 7.4 Fisher Scientific (Waltham, MA, USA). All solvents used were of liquid chromatography-tandem mass spectrometry (LC/MS/MS) grade. Phospholipids NOF cooperation (White Plains, NY, USA). Cholesterol Sigma–Aldrich (Milwaukee, WI, USA). Vancomycin hydrochloride Enzo (VWR Enzo Life Science, San Jose, CA, USA). It had a purity of 99.3%. Vancomycin is soluble in water at a concentration of more than 100 mg/mL, moderately soluble in methanol, and insoluble in acetone, ether, and chloroform.

- Preparation of PEG-VANCO-lipo

Thin-film hydration followed by the freeze–thaw method was used to prepare PEGylated liposomes loaded with vancomycin and was as described elsewhere [14]. The molar ratio of DSPC:PEG-DSPE2000:Cholesterol was 1.85:0.15:1. In brief, phospholipids were weighed out on an analytical balance. They were dissolved in a 50:50 mixture of methanol and chloroform. Thereafter, the solution was subjected to evaporation of solvents using a Rotavac (Buchi, Essen, Germany) with a water bath kept at 55 °C. The lipid film was further dried using nitrogen gas for up to 2 h to ensure complete drying from organic solvents. The film was then hydrated with vancomycin solution in phosphate buffer saline (PBS) pH 7.4 at a concentration of 100 mg/6 mL, which gave rise to crude liposomes. The crude liposomes were then subjected to five cycles of freeze–thaw in liquid nitrogen followed by room temperature. They were then extruded on an extruder (Lipex Northern Lipids, Burnaby, Canada) by gradually reducing the size from 800, 600, 400, and then to 200 nm using membrane filters. The liposomes were controlled for quality based on liposomal particle size in nanometers and polydispersity index and drug content, and encapsulation efficacy [14].

### 2.1. Characterization of Liposomes

#### Dynamic Light Scattering (DLS) Measurements

The Malvern Zetasizer Nano (Malvern Instruments Ltd., Worcestershire, UK) kept at 25 °C was used to measure the average hydrodynamic diameter and polydispersity index (PDI) of the liposome dispersions using an argon–ion laser (488 nm) operating at 10.4 mW using non-invasive backscatter optics (NIBS). The measurement was determined by using dynamic light scattering. Briefly, 20 μL of the liposomal dispersion was diluted to 3 mL of PBS pH 7.4 in a cuvette. This was done to ensure that the liposomes were sufficiently diluted for analysis. The viscosity and refractive index of water were used for data analysis. Prior to this, the system was calibrated with a polystyrene dispersion containing particles of 100 nm. The PDI is a measure for variation in particle size within a liposome population and varies from 0 (complete monodispersity) to 1 (large variations in particle size), and was calculated according to the method of Zhao et al. [29].

### 2.2. Zeta Potential Measurements 

The electrophoretic mobility measurements were done using Zetasizer Nano-Z (Malvern Instruments, UK). The same dilution of liposomes used for DLS measurements was used to perform the zeta potential measurements in PBS pH 7.4. Prior to this, the zetasizer was calibrated using polystyrene latex beads with a defined zeta potential value. The sample dilution was transferred to a disposable folded capillary cell (DTS1070) (Malvern, UK) for zeta potential measurements. 

#### 2.2.1. Determination of Loading Efficiency of VHCL into Liposomes by HPLC 

HPLC analysis was done to determine the amount of VHCL loaded in liposomal dispersion. Briefly, a standard curve was plotted using known concentrations of VHCL. A total of 15 mg of VHCL was dissolved in 10 mL of a mixture of DI water and methanol (1:1) to get a stock solution of 1.5 mg/mL. From this stock solution, subsequent dilutions of 2, 5, 10, 20, 30, 40, and 50 μg/mL VHCL in water for HPLC were prepared. Agilent 1200 high-pressure liquid chromatography (HPLC) with ChemStation software (version Rev. B. 04.03) was used to analyze the content of VHCL. For this HPLC method, a Kinetex Biphenyl column 2.6 mm, 50 × 3 mm column (Phenomenex, Torrance, CA, USA) was utilized. The gradient of acetonitrile (VWR International, PA) was used from 0% to 30% within 5 min. VHCL was eluted with 0.1% formic acid [30]. The wavelength of detection for VHCL was 198 nm. To calculate the loading efficiency, the concentration of VHCL added during the formation of the liposomes (100 mg/6 mL) and the actual concentration of VHCL as obtained in the liposome suspension after extrusion using HPLC was plugged into the following equation:

$$Percentage\;Loading=\left(\frac{Actual\;concentration\;in\frac{\mathrm{mg}}{\mathrm{mL}}as\;determined\;by\;HPLC\;}{Theoretical\;Concentration\;in\;\mathrm{mg}/\mathrm{mL}}\right)\times 100$$

#### 2.2.2. Determination of Encapsulation Efficiency of VHCL into Liposomes by HPLC

Ultracentrifugation (Beckman Coulter) was used to determine the amount of VHCL encapsulated within liposomes. A 500 µL of liposomal dispersion was centrifuged for 30 min at 186,000× *g* at 4 °C, after which the pellet and the supernatant were separated. This was then re-suspended in PBS pH 7.4. In order to lyse the liposomes and liberate VHCL from the pellet, the pellet was dissolved in methanol and mixed thoroughly. HPLC analysis was used to determine the content of VHCL in the supernatant and pellet, as described above. The concentration of VHCL in the pellet and the loading efficiency were plugged into the following equation to obtain the encapsulation efficiency.

$$Percentage\;Encapsulation=\left(\frac{Concentration\;in\frac{\mathrm{mg}}{\mathrm{mL}}of\;VHCL\;in\;the\;pellet}{Loading\;concentration\;in\frac{\mathrm{mg}}{\mathrm{mL}}of\;VHCL}\right)\times 100$$

- Animal Preparation

Animals (*n* = 6/group) were initially anesthetized with ketamine (100 mg/kg) and xylazine (10 mg/kg) by intramuscular (IM) injection. Bupivacaine (0.25%, 0.2 mL/rat) was given subcutaneously (SC) on the surgical site, and animals were monitored 4–6 h post-surgery for postoperative pain control. Bupivacaine injections were repeated if signs of pain were observed. The repeat dose did not exceed 8 mg/kg. A small ventral-middle incision was performed in the neck region. The subcutaneous fat and connective tissue were dissected to expose the right and left jugular veins. Both veins were cannulated with a PU40 tube. Briefly, a small insertion point was rendered by micro-scissors, and the catheter was advanced. The catheter was placed 2–3 cm into the left jugular vein to be in the vena cava. The catheter was secured in the anterior facial branch of the left jugular vein. After fixing the catheter, it was flushed with heparinized saline to verify patency and sealed with stainless steel ring. The left jugular vein was exposed and catheterized. The distal ends of both catheters were tunneled SC to the dorsal scapular region. The left catheter was used for drug administration, and the right catheter was used for blood sampling. PEG-VANCO-lipo or Vancomycin hydrochloride (in PBS) 150 mg/kg was administered IV with a 3 min infusion period to groups of three male SD rats (300 ± 10 g) once daily for three days (qd x3) through left JV catheter.

- Plasma sample collection from rats (serial sampling)

Plasma samples were collected from three rats per group. Blood aliquots (250 μL) were collected at pre-dose and 0.25, 0.5, 1, 2, 4, and 24 h after the first and the last IV from the right jugular vein catheter in tubes coated with EDTA-K2, mixed gently, then kept on ice and centrifuged at 2500× *g* for 15 min at 4 °C, within 1 h of collection. For control animals, blood was collected by cardiac puncture (after euthanized via pentobarbital at 100 mg/kg, intravenously (IV)). The plasma samples were then harvested and kept frozen at −70 °C until further processing.

- Urine sample collection

The urine samples were collected over 0–2, 2–4, 4–8, and 8–24 h after the first and the last IV using the metabolic cages. The urine volumes were recorded. The urine samples were split into two aliquots and then kept frozen at −70 °C until further processing.

- Kidney sample collection from rats

At the study termination (the last time point of the plasma and urine collection), under terminal anesthesia with ketamine (100 mg/kg) and xylazine (10 mg/kg) via IP, rats were euthanized immediately, and the kidneys were harvested. The kidneys were rinsed with a cold saline solution and blotted with a paper towel. The samples were then kept frozen at −70 °C until further processing.

- Estimation of KIM-1 in urine

The KIM-1 levels in pooled 24 h urine samples were analyzed through commercialized ELISA kits (R&D systems, Minneapolis, MN, USA) according to the manufacturer’s instructions.

- Quantitative analysis of vancomycin (plasma and urine samples)

The plasma and urine samples were processed using protein precipitation and analyzed by LC-MS/MS. The detailed chromatographic conditions, bioanalytical methods, and acceptance criteria are summarized in Table 1.

- Pharmacokinetic analysis

Non-compartmental analyses (NCA) were performed using WinNonlin (Version Phoenix 64 Version 8_3.4.295, Princeton, NJ, USA) using the Lambda Z Calculation Method with the Best Fit approach. The following pharmacokinetic parameters such as half-life: t_1/2_; Initial concentration estimated by back-extrapolation: C_0_; AUC_INF_: Area Under Curve (AUC) from time of dosing extrapolated to infinity, based on the last predicted concentration mean residence time: MRT; and Clearance: CL were estimated. The pharmacokinetic analysis was done at Pharmacolgy discovery service, Eurofins labs, Taipei, Taiwan.

- Statistical analysis

All results were expressed in terms of the mean ± standard deviation (SD). The *t*-test or Tukey’s two-way ANOVA test was used to calculate the statistical significance of differences between groups, using GraphPad Prism, Version 9.3.1. Unpaired *t*-tests were used to compare pharmacokinetic parameters between the treatment groups on day one and day three. Statistics were performed in Stata v.17.0 (StataCorp, College Station, TX), and *p* values < 0.05 were considered statistically significant.

## 3. Results

**Figure 1:**
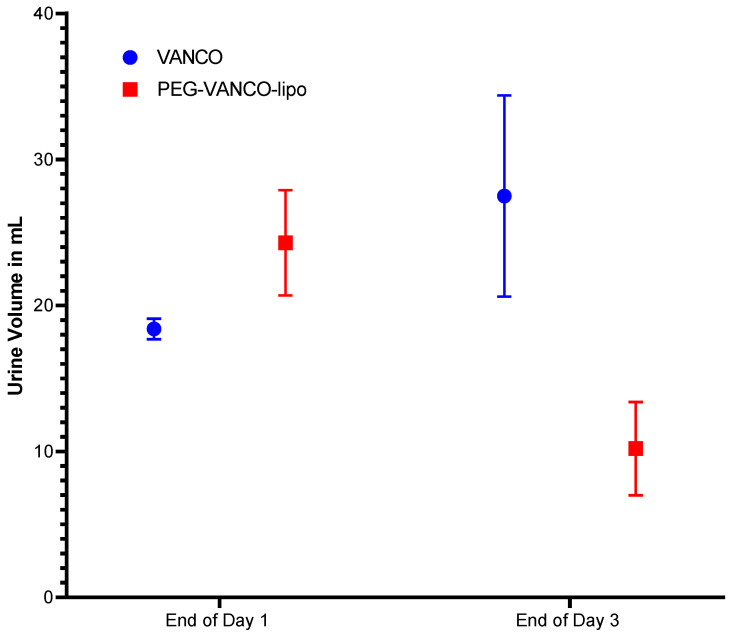
Total rat urine volume at the end of days one and three of treatment with vancomycin hydrochloride in PBS (VANCO) or vancomycin-loaded PEGylated liposomes (PEG-VANCO-lipo) in PBS given intravenously through jugular vein catheter at 150 mg/kg body weight every day for three days, *n* = 6. No statistically significant difference was identified in the two groups when data were analyzed between the two groups at the end of days one and three of the treatment unpaired *t*-test at a 95% confidence interval (*p* > 0.05).

PEG-VANCO-lipo of a diameter less than 200 nm with a polydispersity index (PDI) of less than 0.2 were formulated as reported earlier [14]. A percentage encapsulation of 62% with a loading efficacy of 22.9 mg/mL of vancomycin was observed in PEG-VANCO-lipo after HPLC analysis. The rats’ kidney weight (Table 2) and the urine output volumes are reported in Figure 1. 

No statistical difference was observed in mean kidney weights (*p* > 0.05, Tukey’s two-way ANOVA) of rats from the two treatment groups on day three (Table 2) as well as in total urine output volumes at the end of days one and three (*p* > 0.05, unpaired *t*-test) (Figure 1).

**Figure 2:**
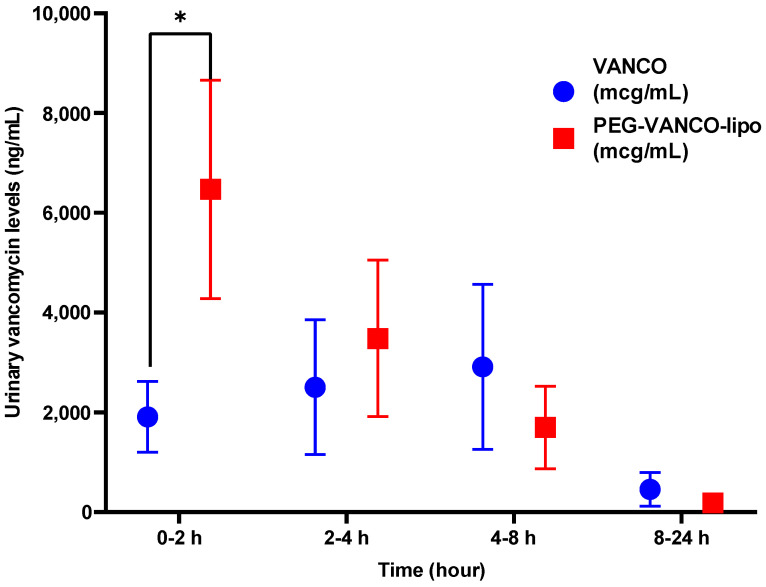
Concentration of vancomycin in rat urine on day three of treatment with vancomycin hydrochloride in PBS (VANCO) or vancomycin-loaded PEGylated liposomes (PEG-VANCO-lipo) in PBS given intravenously through jugular vein catheter at 150 mg/kg body weight every day for three days, *n* = 6. A statistically significant difference was identified using an unpaired *t*-test (*p* < 0.05). * indicates *p* ≤ 0.05.

Almost 3-fold less urinary vancomycin levels were observed for PEG-VANCO-lipo compared to the vancomycin group on day three at the 0–2 h time point and was statistically significant (*p* < 0.05, unpaired *t*-test) (Figure 2). 

**Figure 3:**
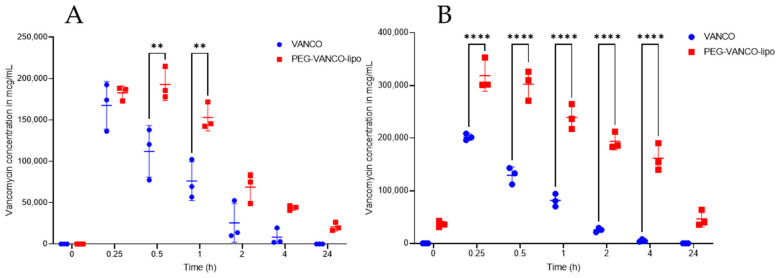
Plasma vancomycin levels (mcg/mL) of rats treated with vancomycin hydrochloride in PBS (VANCO) or PEG-VANCO-lipo in PBS given intravenously through jugular vein catheter at 150 mg/kg body weight every day for three days, *n* = 3, analyzed by LC-MS/MS after sample treatment on day one (**A**) and day three (**B**). Data were analyzed using Tukeys’ two-way ANOVA at a 95% confidence interval (*p* < 0.05). Each animal represents a data point circle or square. ** indicates *p* ≤ 0.01, **** *p* ≤ 0.0001.

The exposure levels (mcg/mL) of vancomycin in rat plasma were determined by liquid chromatography-tandem mass spectrometry (LC-MS/MS) and are shown in Figure 3. There was a 2–5-fold reduction in plasma vancomycin concentration on day one (Figure 3A) at 0.5 and 1 h time points and on day three at 1, 2, and 4 h time points (Figure 3B) *p* ≤ 0.05 in the vancomycin group compared to the PEG-VANCO-lipo group, Tukey’s two-way ANOVA.

**Figure 4:**
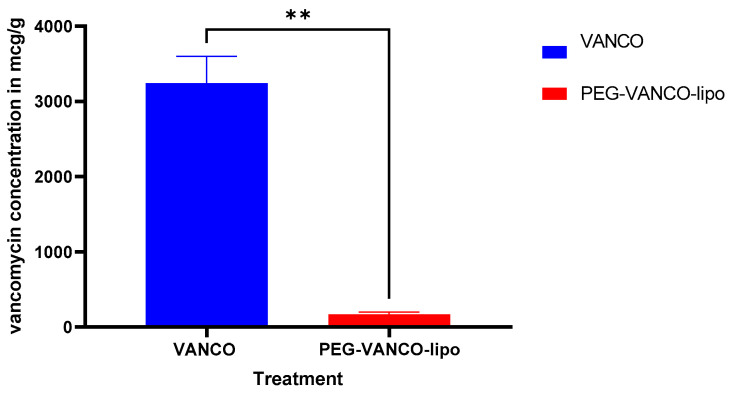
Vancomycin concentration in kidneys (mcg/g) of rats treated with vancomycin hydrochloride in PBS (VANCO) or PEG-VANCO-lipo in PBS given intravenously through jugular vein catheter at 150 mg/kg body weight every day for three days, *n* = 6. Kidneys were minced and analyzed by LC-MS/MS after sample treatment on day three *p* < 0.05 unpaired *t*-test at 95% confidence interval. ** indicates *p* ≤ 0.01.

Almost 20-fold low levels of vancomycin were detected in the PEG-VANCO-lipo group compared to the Vanco group in rat kidneys on day three (*p* < 0.05, unpaired *t*-test) (Figure 4).

**Figure 5:**
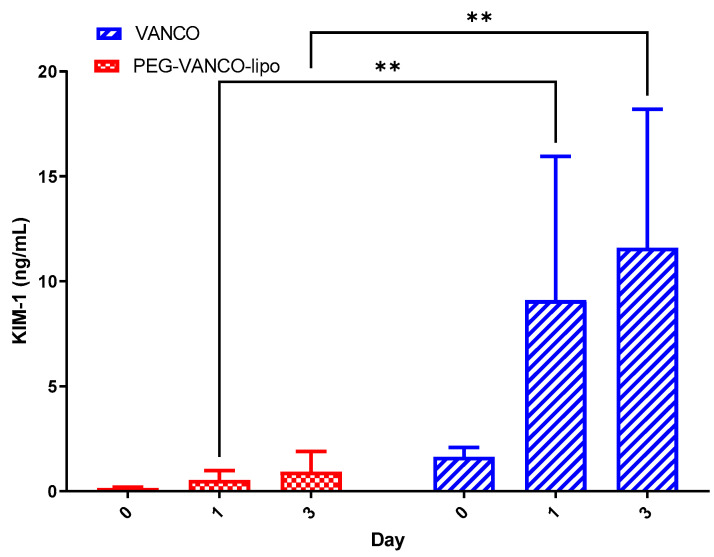
Urinary KIM-1 levels of rats after treatment with 150 mg/kg/day of either vancomycin (VANCO) or PEG-VANCO-lipo over three days, *n* = 6. Data were analyzed using Tukeys’ two-way ANOVA at a 95% confidence interval (*p* < 0.05) ** indicates *p* ≤ 0.01.

Plasma pharmacokinetic parameters for day three for both treatment groups (Vanco vs. PEG-VANCO-lipo) are reported in Table 3. Almost a 2-fold increase in C_0_, 2.5-fold in AUC_INF,_ and a similar reduction in CL were observed in PEG-VANCO-lipo compared to vancomycin (*p* < 0.05, unpaired *t*-test). Similarly, lower urinary KIM-1 levels were detected in the PEG-VANCO-lipo group compared to the vancomycin group and control group (*p* ≤ 0.05 Tukey’s two-way ANOVA). (Figure 5).

## 4. Discussion

The PEG-VANCO-lipo were controlled for quality based on average particle diameter, zeta potential encapsulation, and loading efficiency. The reason for the loading efficiency of 22.9 mg/mL to be higher than the concentration of vancomycin used 100 mg/6 mL (equal to 16.66 mg/mL) during its preparation could be explained due to the loss of buffer during the sizing process. In the animals treated with vancomycin and PEG-VANCO-lipo, no statistical difference was observed in their kidney weights (Table 2). This indicates no additional toxicity because of the encapsulation of vancomycin in liposomes. The total urine output volumes (Figure 1) were mostly similar in both treatment groups at the end of days one and three. The urinary concentration of vancomycin (Figure 2) was higher in animal groups treated with PEG-VANCO-lipo compared to vancomycin on day three only at 0 to 2 h time points. This is indicative of vancomycin being excreted in the kidney at a higher amount in the liposomal formulation at the beginning of the day compared to the vancomycin-treated group, but overall the clearance was found to be decreased in the liposomal formulation. It is indeed a paradoxical finding of higher immediate urinary excretion, yet higher plasma level compared to the free drug that we are not able to explain well. However, the increased urinary vancomycin is seen only at one data point, which is 0–2 h, that could be due to the initial unencapsulated vancomycin getting excreted rapidly or left over from long-circulating liposomes from day one. Correspondingly higher concentration of vancomycin is circulated in plasma from the PEG-VANCO-lipo compared to vancomycin itself both on day one and three (Figure 3A,B), indicating increased AUCI_NF_ (Table 3) and other PK parameters such as increased C_0_ but decreased CL. Other PK parameters, such as t_1/2_ and MRT, had no significant differences (Table 3). 

There was a significantly lower amount of vancomycin accumulated in the rat kidneys of the PEG-VANCO-lipo group than vancomycin (Figure 4), again confirming higher AUC_INF_ results. This also translates into lesser detection of the sensitive kidney injury biomarker (KIM-1) in the PEG-VANCO-lipo group than vancomycin (Figure 5). Based on the results from this experiment, lowering KIM-1 is expected to result in a safer therapy for the kidney. This has been previously supported by the link between KIM-1 and histopathology [9] and glomerular filtration [31,32].

Our results are comparable to previous studies on vancomycin-encapsulated PEGylated liposomes by Muppidi et al. [26], wherein significantly prolonged blood circulation time was observed with reduced accumulation in kidney tissue. However, a detailed comparison between this study [26] and our study cannot be made because of the difference in animal species used (mice vs. rats), the dose administered (5 mg/kg vs. 150 mg/kg), method of analysis (HPLC vs. LC-MS/MS), and probable differences encapsulation efficiency. We hypothesize, based on this and many other studies in the literature, that because of the surface modification via PEGylation, “stealth” liposomes are formed. Polyethylene glycol is a biocompatible hydrophilic polymer. PEG provides a steric hindrance around the liposomes, thus decreasing the opsonization and hence the recognition by the cells of the reticuloendothelial system (RES), resulting in evading the RES. Hence these liposomes are also called “sterically stabilized” liposomes. This, in turn, results in lowering hepatosplenic clearance, thus allowing it to circulate for longer circulation time and hence higher serum concentrations [33]. In addition, this strategy also provides added advantage of increased stability by reducing the interaction with plasma proteins [34]. Many other studies with various different antibiotics (e.g., ciprofloxacin [35], Nafcillin [36], aminoglycosides [37]) have shown that liposomal entrapment might promote the stability and safety of antibiotics, promoting more suitable pharmacokinetic and pharmacodynamic profiles just by extending the circulation time in blood [38,39]. 

Overall, our data indicate that compared to vancomycin in solution alone, PEG-VANCO-lipo reside in the plasma circulation longer. Our toxicity data demonstrate that because of the longer circulation in plasma, kidneys see a lesser accumulation of vancomycin, thus not realizing kidney injury. We hypothesize that because of the longer circulation of PEG-VANCO-lipo in plasma and lesser accumulation in the kidney, toxicity to the kidney is avoided. This preferential distribution may lead to enhanced treatment efficacy clinically, especially with patients with pre-existing impaired renal functions. Further studies are warranted in terms of the biodistribution of vancomycin in other organs, e.g., liver, spleen, lungs, etc., with a histopathological comparison of nephrotoxicity. 

## 5. Conclusions

Vancomycin-loaded PEGylated liposomes resulted in higher AUC, plasma concentrations, and lower clearance circulating for a longer time in the plasma and keeping it away from the kidneys and hence lowering the kidney toxicity as measured by KIM-1. These pharmacokinetic and toxicodynamic data demonstrate great potential for optimizing the safety profile of vancomycin.

## Figures and Tables

**Table 1:** The acceptance criteria for sample analysis.

Sample Type	Criteria for Sample Analysis
Calibration standards (STDs)	The calculated concentrations of the calibration STDs, including the lower limit of quantification (LLOQ) and upper limit of quantification (ULOQ), should not deviate more than 25% from the nominal value (75.0% < Accuracy < 125.0%). At least 75% of the non-zero calibration standards (e.g., 6 in 8 calibration standards) should meet the above criteria.
00 (Double blank) and 0 (Blank)	1. Analyte peak area (00 or 0) ≤ Analyte peak area (LLOQ in calibration curve) 2. IS peak area (00) ≤ IS peak area (LLOQ in calibration curve)
Quality control (QC)	The calculation of the QC samples should be within 25% of the nominal values (75.0% < Accuracy < 125.0%). At least 2/3 of the QC samples should be within the above limits.
Unknown sample	1. The analytical concentrations in the unknown samples were below the 75% LLOQ; they were 0. 2. The analytical concentrations in the unknown samples were above the ULOQ; they were coded “AU” (above the curve limit). The original samples were then diluted with the appropriate matrix and analyzed again in a separate run.

**Table 2:** Rat kidney weight on various days of treatment with vancomycin hydrochloride in PBS or PEG-VANCO-lipo in PBS given intravenously through jugular vein catheter at 150 mg/kg body weight every day for three days, *n* = 6. No statistically significant differences were identified in the two groups using Tukey’s’ 2 way ANOVA at 95% confidence interval (*p* > 0.05).

Treatment		Kidney (g)	
		Left	Right
Vancomycin Hydrochloride (VANCO) (*n* = 6)	Mean	1.158	1.166
	SEM	0.012	0.024
Vancomycin loaded PEGylated liposomes (PEG-VANCO-lipo) (*n* = 6)	Mean	1.127	1.116
	SEM	0.044	0.043
Control (*n* = 1)	Mean	1.375	1.280

**Table 3:** Pharmacokinetic parameters based on vancomycin concentrations on day three of treatment with vancomycin hydrochloride in PBS or PEG-VANCO-lipo in PBS given intravenously through jugular vein catheter at 150 mg/kg body weight every day for three days, *n* = 6. Significant differences were identified in the two groups, especially on day three using an unpaired *t*-test at a 95% confidence interval (*p* < 0.05) ns indicates *p* > 0.05; * indicates *p* ≤ 0.05; ** indicates *p* ≤ 0.01.

Treatment	Vancomycin Hydrochloride (VANCO)		Vancomycin-Loaded PEGylated Liposomes (PEG-VANCO-Lipo)		Unpaired *t*-Test
	Mean	SD	Mean	SD	
t1/2 (h)	13.26	4.85	10.79	1.73	ns
C_0_ (mcg/mL)	186.69	12.22	354.26	91.66	*
AUC_INF_ (h × mcg/mL)	1461.44	285.6	3698.86	837.24	*
MRT (h)	17.073	5.95	12.95	2.7	ns
CL (mL/min/kg)	1.7533	0.32	0.7	0.14	**

## Data Availability

The data presented in this study are available upon request from the corresponding author.
